# Upper gastrointestinal bleeding due to gastric stromal tumour: a case report

**DOI:** 10.1186/1757-1626-3-58

**Published:** 2010-02-12

**Authors:** Tarun Singhal, Sudeendra Doddi, Tessa Leake, Srikanth Parsi, Abdulzahra Hussain, Aninda Chandra, Frank Smedley, Joe Ellul

**Affiliations:** 1 Department of General Surgery, Princess Royal University Hospital, Farnborough, Greater London, BR6 8ND, UK

## Abstract

**Introduction:**

Gastro-intestinal stromal tumours are the most common mesenchymal tumours of the gastro-intestinal tract. This case report highlights the necessity of early surgical intervention in such cases to avoid mortality due to rebleeding and to raise the awareness of rare causes of upper gastrointestinal bleed and their management.

**Case presentation:**

A 61-year-old male presented to the accident and emergency department with a one-day history of haemetemesis with coffee ground vomiting. After initial resuscitation, he underwent upper gastrointestinal endoscopy under sedation which demonstrated a large, bleeding, gastric mass with a central crater along the greater curvature of the stomach. A partial gastrectomy was performed taking a wedge of the stomach with clearance from the tumour, with no signs of extraperitoneal disease.

**Conclusion:**

Early surgical intervention, either open or laparoscopic resection, is the treatment of choice to prevent rebleeds. In general, complete surgical resection is accomplished in 40-60% of all gastro-intestinal stromal tumours patients, and in >70% of those with primary non- metastatic gastro-intestinal stromal tumour. In our case we had completely excised the tumour. Following surgery, all patients must be referred to centres which have more experience in treating gastro-intestinal stromal tumours. Imatinib is proven to be the first effective systemic therapy in cases of unresectable or metastatic disease. All gastro-intestinal stromal tumours have the potential for aggressive behaviour with the risk being estimated from tumour size and mitotic count.

## Introduction

Gastro-intestinal stromal tumours (GIST) are the most common mesenchymal tumours of the gastro-intestinal tract (GI). They account for approximately 0.1 to 3% of all GI neoplasms. Usually, GISTs present in middle age people with a peak age of presentation at 58 yrs, affecting males and females equally. They rarely occur in children or young adults, but when they do, an association with neurofibromatosis and Carney's triad (gastric stromal tumor, extra adrenal paraganglioma and pulmonary chordoma) has been noted [1]. Most frequently, they are solitary, well circumscribed tumors with a pseudocapsule. They arise from embryological mesoderm of the GI tract and were initially thought to be smooth muscle tumours. They are however resistant to chemotherapeutics and have morphological and immunohistochemical features dissimilar to smooth muscle. About 40-70% occur in the stomach, 20-40% in the small intestine, and 5-15% else where in the GI tract (oesophagus, rectum, omentum, peritoneum). Upper GI bleeding is the commonest presentation of GISTS- varying between 50-100% depending on the case series. However, GISTs as a cause of upper GI bleeding is rare. They can also present with abdominal pain, dyspepsia and vomiting or incidental findings during endoscopy, imaging or surgery.

The clinical presentation of GISTs is variable. It depends on the size and organ involvement. Gastric GISTs usually present with vague abdominal pain, dyspepsia, and vomiting. They rarely present with secondary complications such as upper GI bleeding and perforation. Asymptomatic GISTs are found incidentally during surgery, endoscopy or CT scan for other conditions.

This case report highlights the necessity of early surgical intervention in such cases to avoid mortality due to rebleeding and to raise the awareness of rare causes of upper GI bleed and their management.

## Case presentation

**Figure 1:**
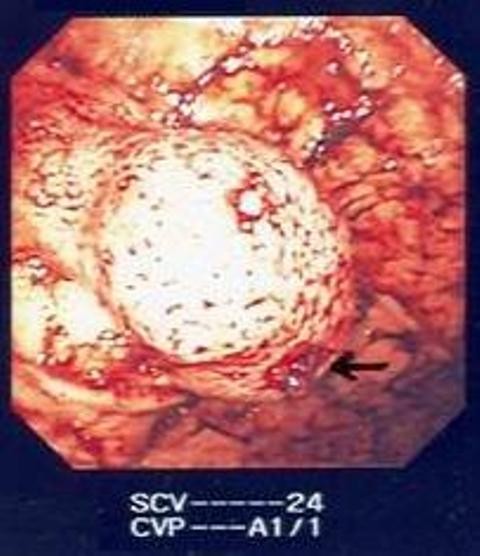
**Gastric mass with a bleeding vessel in a central crater**.

**Figure 2:**
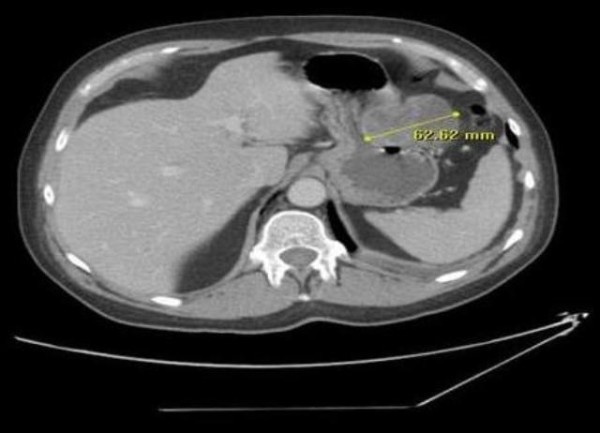
**CT scan revealing 6 cm mass with no evidence of extraperitoneal spread**.

**Figure 3:**
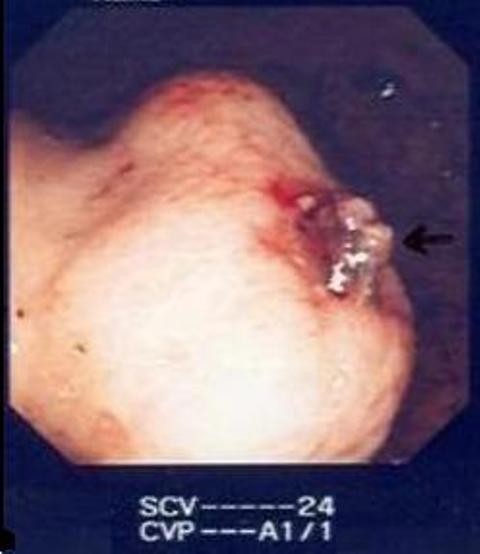
**Bleeding vessel after control**.

A 61-year-old British Caucasian male presented to the Accident and Emergency with a one-day history of haemetemesis with coffee ground vomiting. He was an ex-alcoholic with no history of chronic liver disease or peptic ulceration. After initial resuscitation, he underwent upper GI endoscopy under sedation which demonstrated large clots in the stomach (Figure 1). Repeat upper GI endoscopy was therefore performed urgently under general anaesthesia with a therapeutic endoscope. A large, bleeding, gastric mass with a central crater was identified along the greater curvature of the stomach after the aspiration of clots. Bleeding was then controlled with the use of adrenaline injection and argon plasma coagulation. He needed a blood transfusion following the procedure. Following this, he had a CT scan which showed a well-defined, dumb-bell shaped 6 cm mass arising from the greater curvature of stomach with most of the mass being extra luminal (Figure 2). There was no evidence of distant lesions. He had another episode of haematemesis 48 hours after the therapeutic endoscopy necessitating 5 units of blood transfusion. It was therefore decided to proceed with an upper GI endoscopy (Figure 3) with a view to proceed to immediate laparotomy and resect the tumor, which was the source of the rebleeding.

**Figure 4:**
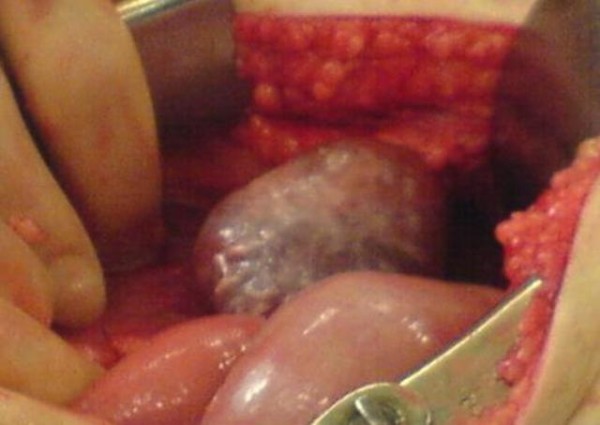
**Intra-operative image showing GIST before resection**.

At laparotomy, the tumour was identified on the greater curvature with no evidence of liver, peritoneal, omental or lymph nodal lesions. A partial gastrectomy was performed taking a wedge of the stomach with clearance from the tumour (Figure 4).

**Figure 5:**
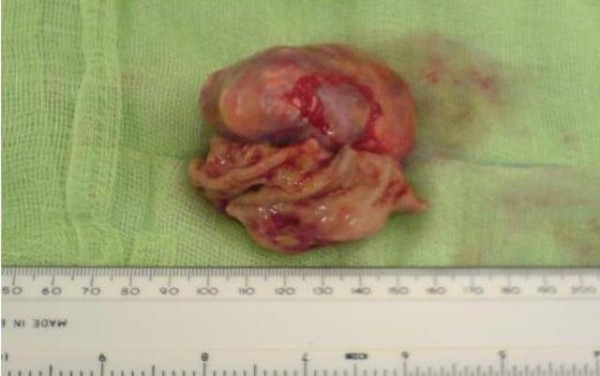
**Highlighting the GIST specimen once removed post operatively measuring 6 cm**.

**Figure 6:**
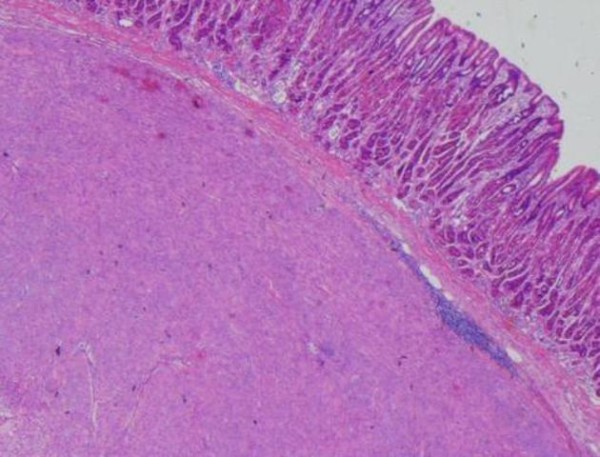
**Histopathology specimen highlighting low mitotic activity**.

**Figure 7:**
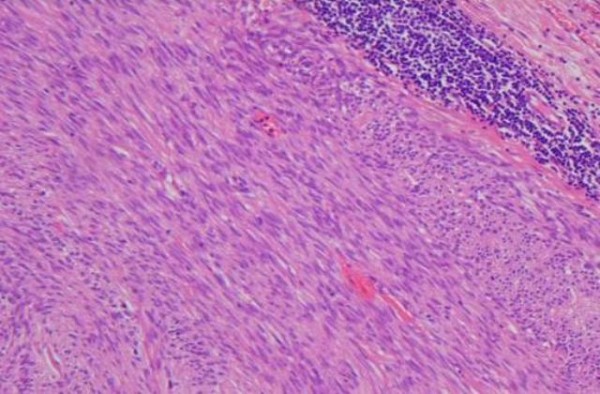
**Specimen showing spindle cells with no significant nuclear pleomorphism**.

**Figure 8:**
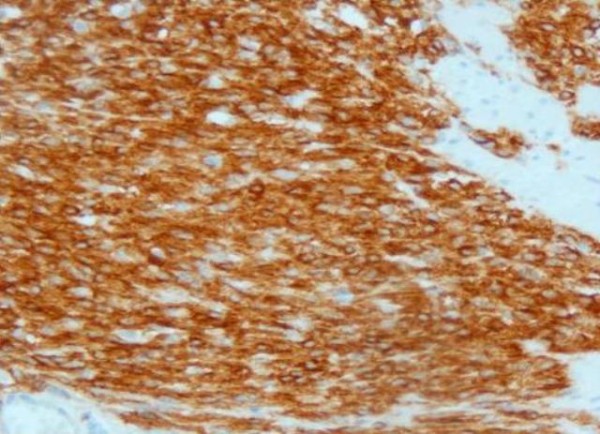
**Showes immunostaining of the tumor cells which were strongly positive for CD117 and negative for S100, desmin, smooth muscle, and actin**.

The histology report showed a 7 cm (Figure 5), well circumscribed, non-encapsulated tumour within submucosa and muscularis propria. On microscopy the tumour was composed of spindle cells with no significant nuclear pleomorphism (Figure 6). The mitotic count was low (less than 5 mitosis per 50 HPF) with no evidence of dysplasia or malignancy of overlying gastric mucosa (Figure 7). Immunostaining of the tumor cells were strongly positive for CD117 and negative for S100, desmin, smooth muscle, and actin (Figure 8). The above features strongly suggested the diagnosis of GIST. He did well following surgery. Subsequently he was entered into a randomised controlled trial for Glivac.

## Discussion

The incidence of GISTs has increased in the last few years due to better detection as all mesencymal tumours are now being tested for CD117. CD117 (Kit protein) is the product of c-kit proto-oncogene, located on chromosome 4q11-21. This protein is a tyrosine kinase growth factor receptor present in 90% of GIST cells. Mutation of kit proto-oncogene results in a CD117 receptor that is constitutively stimulated without the presence of the stem-cell growth factor [2]. Some of the GISTs that lack the kit mutation appear to have a mutation in another Class III protein kinase gene that encodes the platelet-derived growth factor [3]. It is now believed that these tumours arise either from stem cells that differentiate towards interstitial cells of Cajal (these cells form part of the myenteric plexus in the gastrointestinal tract and regulate peristalsis) or directly from interstitial cells of Cajal and not from smooth muscle cells [4]. The annual incidence of GIST is 15 per million and the prevalence is approximately 130 per million in western populations.

GIST is an unusual cause of Upper GI bleed, and has a high propensity to rebleed. These bleeding tumours need to be investigated urgently as an inpatient rather than as an outpatient. Early surgical intervention, either open or laparoscopic resection, is the treatment of choice to prevent rebleeds. In general, complete surgical resection is accomplished in 40-60% of all GIST patients, and in >70% of those with primary non- metastatic GIST [5]. In our case we had completely excised the tumour.

GISTs exhibit a highly variable behaviour after resection of the primary tumour. These patients need to be followed up on a long term basis as local recurrence and metastases can occur many years after surgery. These tumours spread by the haematogenous route predominantly to the liver. Lymph node involvement in very rare and therefore lymphadenectomy is not routinely indicated [6]. In general, local recurrence or metastases develop in approximately 50% of patients who had potentially curative operation [7]. The median disease specific survival for patients with primary GIST is approximately 5 years [8]. The two most important tumor factors for local recurrence and metastasis are tumour size and mitotic rate (size >5 cms and mitosis >5 per 50 HPF increases the risk) [9]. Other prognostic factors are completeness of resection, age, and tumor location. Gastric GISTs have a lower risk of tumor recurrence than oesophageal, small bowel or large bowel GISTs.

Before the Imatnib era surgical resection was the only option available as GIST are highly resistant to chemotherapy and radiotherapy. The 5 year survival rate was 35-65% following complete resection and the medial survival rate was 10-20 months for unresectable disease. The introduction of Imatinib mesylate, a tyrosine kinase inhibitor, has dramatically improved the outcomes of treatment. It had demonstrated in clinical trials a significant decrease in tumour size rendering initially inoperable tumour resectable [10]. In phase I and II trials, Van Ooseterom et al [10] and Demetri et al, [11] saw a partial response (reduction of at least 50% of tumour burden) in 79% of patients, stable disease in 28%, and progressive disease in 13%. The overall survival rate after 1 year was 88%, although the median duration of survival is yet to be defined. Currently imatinib is approved only for the treatment of advanced disease. The recommended starting dose is 400-600 mgs. Recent European phase III trails have demonstrated that 400 mgs twice daily significantly prolonged progression free survival. It is well tolerated orally with minor side effects like rash, nausea, diarrhoea, muscle cramps, periorbital and peripheral oedema. Myelosuppression was rare. The duration of treatment has not yet been defined. The phase II trials of Radiation and oncology Group study S-0132 recommends imatinib for 2 years [12].

The current recommendations in management of GIST are as follows; [13]

• For operable GISTs, perform surgery first followed by adjuvant therapy with Imatinib in high risk patients (size > 5 cms, mitotic rate >5 per 50 HPF, incomplete resection, tumour spillage).

• For marginally resectable GIST or in case of inoperable recurrent or metastatic GIST, consider neoadjuvant therapy with Imatinib followed by surgical resection.

• For intermediate risk GIST (size < 5 cms and 6-10 mitosis per 50 HPF, or 5-10 cms and, 5 mitosis per 50 HPF), the role of Imatinib as an adjuvant therapy is still debatable.

Follow up guidelines following resection of GIST are yet to be defined for low risk, intermediate risk and high risk GIST. According to National Comprehensive Cancer Network practice guidelines all completely resected GIST are followed in clinic with CT scan every 3-6 month for the first 5 years and then annually. Less surveillance is acceptable for low risk patients [14].

## Conclusion

GIST is an unusual cause of upper GI bleeding. Early surgical intervention is the treatment of choice to prevent rebleeds. All suspected mesenchymal tumors of GI should be tested for CD117 by an experienced histopathologist. Following surgery, all patients must be referred to centres which have more experience in treating GIST. Imatinib has proven to be the first effective systemic therapy in cases of unresectable or metastatic disease. In case of operable GIST, Imatinib is indicated as an adjuvant therapy in high risk patients. All GISTS have the potential for aggressive behaviour, the risk being estimated from tumour size and mitotic count [15].

## Consent

Written informed consent was obtained from the patient for publication of this case report and accompanying images. A copy of the written consent is available for review by the Editor-in-Chief of this journal.

## Competing interests

The authors declare that they have no competing interests.

## Authors' contributions

TS and SD prepared case report, PS, TL, AC analysed case report and performed literature search, JE and FS identified case and performed discussion of case. All authors read and reviewed the final manuscript.
